# Comparative transcriptome profiling reveals a network of differentially expressed genes in Asia II 7 and MEAM1 whitefly cryptic species in response to early infection of *Cotton leaf curl Multan virus*

**DOI:** 10.3389/fmicb.2022.1004513

**Published:** 2022-10-04

**Authors:** Tahir Farooq, Qi Lin, Xiaoman She, Ting Chen, Yafei Tang, Zifu He

**Affiliations:** Plant Protection Research Institute and Guangdong Provincial Key Laboratory of High Technology for Plant Protection, Guangdong Academy of Agricultural Sciences, Guangzhou, China

**Keywords:** *Cotton leaf curl Multan virus*, *Bemisia tabaci*, RNA-seq, virus-vector interaction, comparative transcriptomics, DEGs, immune responses

## Abstract

*Cotton leaf curl Multan virus* (CLCuMuV) is a whitefly-vectored begomovirus that poses ramping threat to several economically important crops worldwide. The differential transmission of CLCuMuV by its vector *Bemisia tabaci* mainly relies on the type of whitefly cryptic species. However, the molecular responses among different whitefly cryptic species in response to early CLCuMuV infection remain elusive. Here, we compared early-stage transcriptomic profiles of Asia II 7 and MEAM1 cryptic species infected by CLCuMuV. Results of Illumina sequencing revealed that after 6 and 12 h of CLCuMuV acquisition, 153 and 141 genes among viruliferous (VF) Asia II 7, while 445 and 347 genes among VF MEAM 1 whiteflies were differentially expressed compared with aviruliferous (AVF) whiteflies. The most abundant groups of differentially expressed genes (DEGs) among Asia II 7 and MEAM1 were associated with HTH-1 and zf-C2H2 classes of transcription factors (TFs), respectively. Notably, in contrast to Asia II 7, MEAM1 cryptic species displayed higher transcriptional variations with elevated immune-related responses following CLCuMuV infection. Among both cryptic species, we identified several highly responsive candidate DEGs associated with antiviral innate immunity (alpha glucosidase, LSM14-like protein B and phosphoenolpyruvate carboxykinase), lysosome (GPI-anchored protein 58) and autophagy/phagosome pathways (sequestosome-1, cathepsin F-like protease), spliceosome (heat shock protein 70), detoxification (cytochrome P450 4C1), cGMP-PKG signaling pathway (myosin heavy chain), carbohydrate metabolism (alpha-glucosidase), biological transport (mitochondrial phosphate carrier) and protein absorption and digestion (cuticle protein 8). Further validation of RNA-seq results showed that 23 of 28 selected genes exhibited concordant expression both in RT-qPCR and RNA-seq. Our findings provide vital mechanistic insights into begomovirus-whitefly interactions to understand the dynamics of differential begomovirus transmission by different whitefly cryptic species and reveal novel molecular targets for sustainable management of insect-transmitted plant viruses.

## Introduction

Of approximately 1,100 plant pathogenic viruses that are recognized by the International Committee on Taxonomy of Viruses (ICTV), over 75% are vectored by different insect species, mainly by whitefly (*B. tabaci*) (Hogenhout et al., 2008; Ng and Zhou, 2015). *B. tabaci* is capable of inducing substantial crop losses via direct feeding and vectoring different plant viruses (Brown et al., 1995). The whitefly-vectored DNA plant viruses are devastating agricultural pathogens that cause serious economic crop losses all over the world. With more than 445 species (Walker et al., 2020), begomoviruses of the family *Geminiviridae* represent the largest group of single-stranded DNA (ssDNA) viruses that are well-known for whitefly-mediated transmission. These begomoviruses are exclusively transmitted by *B. tabaci* species complex in a persistent circulative manner (Harrison et al., 2002; Rosen et al., 2015). To date, ∼45 *B. tabaci* cryptic species have been reported worldwide (Rehman et al., 2021), among which, the Middle East-Asia Minor 1 (MEAM1, previously B biotype) is widely distributed and the most abundant cryptic species in several regions of the world (De Barro et al., 2011) and a number of begomoviral outbreaks have been associated with MEAM1 outbreaks (Varma and Malathi, 2003; Colvin et al., 2006; Jiu et al., 2007). In China, the first invasion of MEAM1 was reported in the (mid to late) 1990s (Chen et al., 2002). Moreover recently, another *B. tabaci* cryptic species Asia II 7 (also called CV biotype) has been reported to efficiently transmit a begomovirus (*Cotton leaf curl Multan virus*, CLCuMuV) infecting malvaceous crops in China (Chen et al., 2019).

The virus-vector interactions are vital for both the population dynamics of insect vectors and the dissemination of plant viruses (Stout et al., 2006). Owing to their known importance, whiteflies and begomoviruses have been widely used as models to investigate plant-virus-vector interactions (Götz et al., 2012; Rana et al., 2016; Roy et al., 2021). The begomoviruses are acquired by their vector whiteflies followed by their movement from the gut lumen into the hemolymph and then to the salivary glands from where virions are secreted into the host plant tissues during the insect feeding process (Czosnek et al., 2002; Hogenhout et al., 2008). These begomovirus-whitefly interactions are complex and the direct or indirect impact of these viruses can induce different responses in the insect vectors. Recently, several studies have reported different transcriptomic responses induced by begomoviruses in their whitefly vectors. For example, comparative transcriptomic profiling of MEAM1 whiteflies infected with a begomovirus (Tomato yellow leaf curl virus, TYLCV) and a crinivirus (Tomato chlorosis virus, ToCV) reveals a number of transcription factors, receptors, cathepsins and hemocyanin genes involved in regulation of the insect antiviral immunity along with their possible role in the virus transmission (Hasegawa et al., 2018). Another study describes the transcriptomic changes of Asia II 1 whiteflies after infection with a begomovirus (Chilli leaf curl virus, ChiLCV) revealing several candidate genes associated with virus infection and its circulative movement in the insect vector (Nekkanti et al., 2022). Likewise, the transcriptional changes in the guts of TYLCV-infected MEAM1 whiteflies showed that TYLCV could activate insect immune responses and several other genes associated with intracellular signaling (Geng et al., 2018).

CLCuMuV along with a betasatellite (Cotton leaf curl Multan betasatellite, CLCuMuB) is a whitefly-vectored begomovirus that was originally reported as the major pathogen associated with cotton leaf curl disease (CLCuD) in Multan, Pakistan (Briddon and Markham, 2000; Mansoor et al., 2003). Since then, the virus has rapidly spread to major cotton-growing countries, particularly in South Asia. A recent study highlights the geographical distribution and standing genetic diversity of highly recombinant and rapidly evolving CLCuMuV populations across major cotton-producing countries (Farooq et al., 2021). Although local transmission of CLCuMuV is attributed to the whitefly cryptic species complex, the source of virus spread from Pakistan to China or other countries has not been well-established (Ashfaq et al., 2014). In China, Asia II 7 and MEAM1 whitefly cryptic species were observed to infest the diseased plants displaying CLCuD symptoms (Ting et al., 2016). Although a comparative study demonstrates that CLCuMuV is efficiently transmitted by Asia II 7 while MEAM1 is incompetent to transmit the virus, it was observed that MEAM1, MED and Asia II 7 cryptic species were able to acquire the virus as early as 6 h post feeding on the virus-infected plants (Chen et al., 2019).

In efforts to investigate the interaction mechanisms between whiteflies and begomoviruses, several transcriptomics-based studies have been performed to analyze the molecular responses of whiteflies to diverse begomoviruses (Luan et al., 2011, 2013; Kaur et al., 2017; Geng et al., 2018; Hasegawa et al., 2018; Ding et al., 2019; Li et al., 2020). However, most of the previously reported studies mainly focus on the MEAM1 whiteflies and to our knowledge, no study has compared the molecular responses of Asia II 7 and MEAM1 cryptic species after CLCuMuV infection. Notably, the transcriptional changes in response to CLCuMuV at the early stages of virus infection remain unknown. Here, we employed Illumina sequencing to map and compare these transcriptional responses among Asia II 7 and MEAM1 cryptic species following acquisition access period (AAP) of 6 and 12 h. We have identified several candidate differentially expressed genes (DEGs) that might play vital roles in the regulation of the differential transcriptomic responses during CLCuMuV-whitefly interactions. Our results will further expand the current knowledge of whitefly-virus interactions by providing new insights into the early molecular responses of begomovirus-infected whiteflies.

## Materials and methods

### Insect culture, virus inoculum, and plants

*Bemisia tabaci* Asia II 7 and MEAM1 populations were maintained on cotton (*G. hirsutum* cv. Zhongmian 43) seedlings as this cultivar is a non-host plant of CLCuMuV (Tang et al., 2017). The purity of *B. tabaci* Asia II 7 (biotype CV) and MEAM1 (biotype B) cryptic species cultures was confirmed every 3-5 generations by using mitochondrial cytochrome oxidase I (*mtCOI*) gene-specific primers mtCOI-C1- J- 2195/mtCOI-TL2N- 3014 (Supplementary Table 1). For cryptic species purity analysis, 10-15 individual whiteflies were randomly sampled, subjected to PCR analysis and confirmed by sequencing. The whitefly cultures were maintained in the climate chamber with 14-h light/10-h dark photoperiod at a temperature of 27 ± 1*^o^*C and relative humidity of 70 ± 10%.

The CLCuMuV was maintained by infiltrating the *G. hirsutum* cv. Xinhai 21 at 2-3 true-leaf stage with Agrobacterium GV3101 strain cultures harboring CLCuMuV DNA-A (KP762786) and betasatellite (KP762787). The presence of CLCuMuV in plants was confirmed by the development of typical symptoms and by PCR testing using specific primers CLCuMuV-CL F/R & CLCuMuB-beta-F/R (Supplementary Table 1). All plants were grown and maintained in a growth chamber with 14-h light/10-h dark photoperiod at a temperature of 25-27*^o^*C and relative humidity of 60%.

### Virus acquisition assay, sample collection, deoxyribonucleic acid, and ribonucleic acid isolation

Approximately 2,500 adult whiteflies were collected and transferred to CLCuMuV-infected and uninfected plants to obtain VF and AVF whiteflies, respectively. Following the acquisition access period (AAP) of 6 and 12 h, ∼ 300 live VF whiteflies were collected from CLCuMuV-infected plants and AVF whiteflies from uninfected plants. For each treatment, 3 biological replicates were included. The VF and AVF whiteflies were frozen in liquid N and preserved at ultra-low temperature (−80*^o^*C). To confirm the acquisition of CLCuMuV by Asia II 7 and MEAM1 whiteflies, 15 individual AFV whiteflies (after 6 and 12 AAP) were selected and total DNA extraction was performed using lysis buffer following a previously described protocol (Pan et al., 2017). Subsequently, PCR-based detection of CLCuMuV was done using Premix Taq*™* (TaKaRa Taq*™* Version 2.0 plus dye) (Takara, Tokyo, Japan) with specific primers (Supplementary Table 1). The whiteflies fed on uninfected cotton plants were included as negative control. Total RNA was isolated from VF and AVF whiteflies (Asia II 7 and MEAM1 cryptic species) by using TRIzol reagent (Life Technologies, Inc., MD, USA) following the manufacturer’s instructions. A total of 18 samples comprising VF (with 6 and 12 h AAP) and AVF whiteflies (fed on virus-free cotton plants) were subjected to RNA extraction. The quantity and integrity of RNA were analyzed by NanoDrop 2000 spectrophotometer (Thermo Fisher Scientific, CA, USA) and visualization via 0.8% gel electrophoresis, respectively. Additional information on the RNA concentration, OD (230/230 and 260/280) and RNA integrity number (RIN) is given in Supplementary Table 2.

### Preparation of cDNA library, transcriptome sequencing, assembly, and analysis

To generate paired-end cDNA libraries, ∼ 1.5 μg RNA/sample was processed using an ABclonal mRNA-seq Lib Prep Kit (ABclonal, China) following the manufacturer’s protocol and AMPure XP system was used to obtain the purified products. The quality of these libraries was assessed using Agilent Bioanalyzer 4,150 system. Subsequently, these libraries were sequenced in a paired-end mode using Illumina Novaseq 6000/MGISEQ-T7 platform (Illumina BGI, China). The clean data were obtained by removing reads containing adapters, low-quality reads and poly-N-containing reads. The reads were filtered and the high-quality, paired-end reads were aligned to *B. tabaci* reference genome. A python-based framework HTSeq v0.6.1 was employed to count the read numbers mapped to the genes. The expected number of Fragments Per Kilobase of transcript sequence per Million base pairs sequenced (FPKM) of each gene was estimated based on the reads count mapped to that gene and the gene length.

### Differential gene expression analysis

Analysis of the differentially expressed genes between VF and AVF whiteflies (Asia II 7 and MEAM 1 cryptic species) was performed using R package, DESeq2 (Love et al., 2014). The obtained raw *p*-values were then adjusted for multiple testing using false discovery rate (FDR). Further, to identify differentially expressed genes, we used the cut-off FC ratio ≥2 for up-regulated genes and FC ratio ≤ −2 for down-regulated genes with Padj <0.05. The data variability between VF and AVF groups was analyzed by principal component analysis (PCA).

### Gene functional annotation and pathway enrichment analyses

In order to obtain comprehensive gene functional information, we employed five different databases, including Non-supervised Orthologous Groups (NR), Pfam, Swiss-Prot, Kyoto Encyclopedia of Genes and Genomes (KEGG) and Gene Ontology (GO) for annotation of the non-redundant transcripts. The characteristics of these databases, methods, parameters and E-value thresholds used for annotation are given in Supplementary Table 3.

### Coding sequences and transcription factor prediction

The assembly of each RNA-Seq dataset was performed by Trinity software with default parameters. The prediction of coding sequences (CDS) was divided into two steps. First, the Trinity-assembled runs were analyzed using NR and Swiss-Prot databases with an E-value threshold of 1e-5. Then, the results of the comparison were used to extract the coding frame information and then the coding regions were translated into amino acid sequences according to the standard codon table. Second, for the sequences that were not compared or predicted in the NR (threshold *E*-value = 1e-5) or Swiss-Prot libraries (threshold *E*-value = 1e-5), the ORF was predicted by TransDecoder software (Haas et al., 2013) with standard parameters (-S: -m 100; -G universal: -S). To annotate the transcription factors (TFs), we screened and aligned the sequences in the Animal transcription factors database (AnimalTFDB, *E*-value <0.0001) and used the corresponding information to predict TFs of different genes.

### Quantification of the candidate differentially expressed genes by RT-qPCR

To validate the results of DEGs analysis, the mRNA expression profiles of selected 28 genes were quantified by RT-qPCR and normalized against β*-actin* and *elongation factor 1-alpha* as the internal control gene. We categorized the genes selected for RT-qPCR analysis into three groups based on these criteria: (i) their significant indicative functions associated with virus transmission/virus-vector interactions, (ii) based on their significant (low or high) expression patterns, and (iii) randomly selected genes. The RNA extraction was performed following the same method as described above and an aliquot of RNA was taken for RT-qPCR-based validation of the target gene/s expressions. The cDNA synthesis was performed by using HiFiScript gDNA Removal RT Mastermix, CW2020M (CoWin Biosciences, China) as per provided instructions. RT-qPCR was performed using TB Green^®^ *Premix Ex Taq*™ II (Tli RNaseH Plus) (Takara Bio, Inc.) following the manufacturer’s protocol. A total of three biological and nine technical repeats were used in each treatment and the relative mRNA expression was calculated by the previously described 2^–ΔΔCT^ method (Livak and Schmittgen, 2001). Details of the primers used for RT-qPCR experiment are given in Supplementary Table 1.

## Results

### Transcriptomic profiling of viruliferous and aviruliferous whiteflies, data quality, and transcriptome assembly

To determine the early transcriptional responses of CLCuMuV-infected or healthy Asia II 7 and MEAM1 cryptic species at different time intervals (0, 6 and 12 h), we performed RNA-seq analysis. A total of 18 cDNA libraries were subjected to Illumina sequencing which generated 306,659,552 and 602,129,506 raw reads for AVF and VF whiteflies, respectively (Table 1). Subsequently, after cleaning and quality checking 305,700,412 and 600,243,414 clean reads were obtained for AVF and VF whiteflies (Supplementary Figure 1). Results of the sequenced data quality control showed that the average percentage Q20 and Q30 values of the 18 samples were 95.95 and 89.78%, respectively (Table 1). Further, Pearson’s correlation analysis revealed that 18 samples between replicated libraries from different treatments were highly correlated (Supplementary Figure 1). Results of mapping indicated that the clean reads from all treatments were mapped to trinity spliced transcriptomes at percentages ranging between 80.75 and 83.45%. The GC contents ranged between 39.46 and 44.49% (Supplementary Table 4). Results of the unigene transcripts length distribution and NR-based comparison of mapped species demonstrated that with an average length of 743 bp, the highest number (28,786) of unigenes were mapped to *B. tabaci* genome (Supplementary Figure 2).

**TABLE 1:** Statistical summary of RNA-seq libraries from whiteflies (Asia II 7 and MEAM1 cryptic species) fed for 6 and 12 h on CLCuMuV-infected or uninfected (0 h) cotton plants.

Sample	Raw_reads	Clean_reads	Clean_bases	Error (%)	Q20 (%)	Q30 (%)	GC (%)
B0H-1	52531842	52366826	7.81G	0.05	95.75	89.33	41.85
B0H-2	62584888	62397588	9.3G	0.05	95.71	89.23	42.24
B0H-3	43089662	42955154	6.38G	0.05	96.13	90.14	42.47
B12H-1	54972724	54801270	8.12G	0.05	95.69	89.14	42.13
B12H-2	45371378	45232384	6.73G	0.05	95.94	89.78	41.58
B12H-3	47898562	47747744	7.07G	0.05	96.16	90.23	43.07
B6H-1	50024608	49866964	7.42G	0.05	96.15	90.23	40.36
B6H-2	61216346	61026362	9.07G	0.05	96.06	90.02	39.46
B6H-3	55118502	54944528	8.16G	0.05	96.13	90.16	41.32
CV0H-1	51411916	51248146	7.61G	0.05	96.19	90.34	43.6
CV0H-2	50333280	50174214	7.44G	0.05	96.23	90.44	41.08
CV0H-3	46707964	46558484	6.91G	0.05	96.12	90.17	43.05
CV12H-1	48683114	48529992	7.2G	0.05	95.52	88.79	43.02
CV12H-2	43156414	43020410	6.39G	0.05	96.14	90.2	42.74
CV12H-3	58788776	58605038	8.75G	0.05	95.84	89.56	40.5
CV6H-1	50828682	50668854	7.51G	0.05	95.44	88.62	42.86
CV6H-2	44886010	44746026	6.64G	0.05	96.04	90.01	43.51
CV6H-3	41184390	41053842	6.09G	0.05	95.91	89.69	44.49

### Differentially expressed genes among viruliferous and aviruliferous groups of Asia II 7 and MEAM1 whiteflies at different time intervals

**FIGURE 1:**
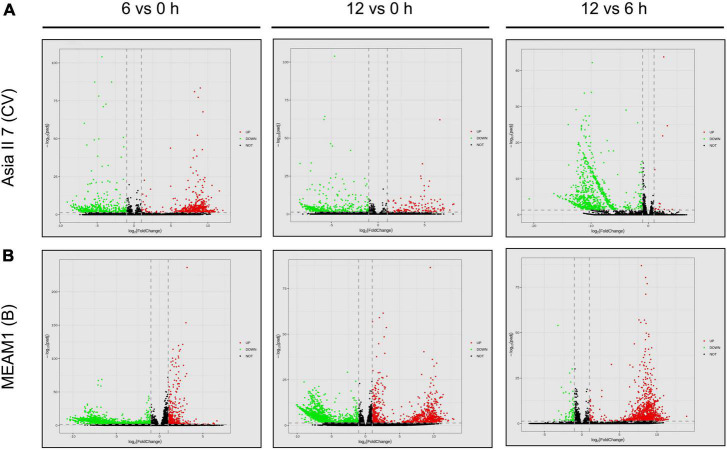
Pairwise comparisons of differentially expressed genes (DEGs) among VF and AVF whiteflies (Asia II 7 and MEAM1). The cut-off FC ratio ≥2 for up-regulated genes and FC ratio ≤−2 for down-regulated genes with Padj <0.05 were used to identify DEGs. DEGs among **(A)** Asia II 7 and **(B)** MEAM1 cryptic species after 6 and 12 h of CLCuMuV infection as compared to those fed on uninfected cotton plants. Each gene is represented by scattered dots in the figure. Red colored dots represent the up-regulated genes, green colored dots indicate down-regulated genes while black color indicates non-significantly expressed genes.

**FIGURE 2:**
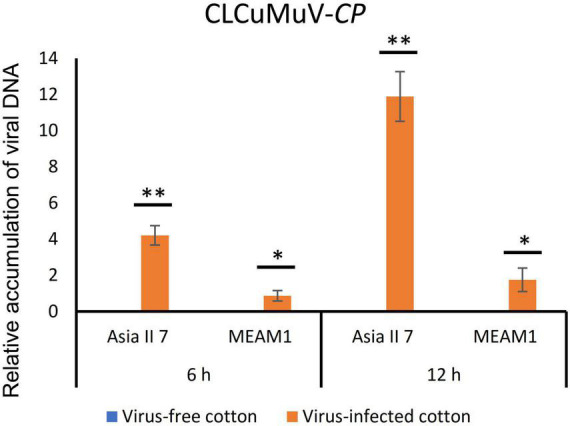
Comparative gene expression analysis among Asia II 7 and MEAM1 cryptic species. The quantification of CLCuMuV-CP was performed using DNA of whiteflies fed on the virus-free and virus-infected *Gossypium hirsutum* cv. Xinhai plants for 6 and 12 h. Student *t*-test was used to analyze the statistically significant differences between viruliferous and aviruliferous whitefly groups. Asterisks denote statistical significance: *P < 0.05; ^**^P < 0.01.

We assessed the global patterns of DEGs in two groups of whitefly cryptic species at 6 and 12 h post-CLCuMuV infection. Among 9 comparative groups of VF whiteflies, a total of 27,174 DEGs were found. Further analysis showed that after 6 and 12 h of CLCuMuV AAP, 153 (12 up-regulated and 141 down-regulated) and 141 (3 up-regulated and 138 down-regulated) genes were differentially regulated in Asia II 7 cryptic species. Whereas, at 12 h of CLCuMuV AAP, 35 (5 up-regulated and 30 down-regulated) genes were differentially expressed among Asia II 7 whiteflies as compared to those fed on CLCuMuV-infected plants for 6 h (Table 2 and Figure 1). On the other hand, among MEAM1 whiteflies, 445 (251 up-regulated and 194 down-regulated) and 347 (171 up-regulated and 176 down-regulated) genes were differentially regulated at 6 and 12 h post-feeding on the CLCuMuV source, respectively. While at 12 h time point, there were only 13 significantly up-regulated and 39 down-regulated genes as compared to those in MEAM1 fed on the virus source for 6 h (Table 2 and Figure 1). Captivatingly, the prevalent proportions of DEGs in the Asia II 7 and MEAM1 whiteflies were associated with suppressed and accelerated groups, respectively (Figure 1). Among Asia II 7 whiteflies, phosphoenolpyruvate carboxykinase (GTP) was significantly downregulated at 12 h AAP followed by alpha-glucosidase, sequestosome-1 isoform X1, protein msta, isoform A and vitellogenin (Supplementary Table 5). On the other hand, cathepsin F-like protease was significantly up-regulated among VF MEAM1 whiteflies at 12 h AAP followed by vitellogenin, an uncharacterized protein, neutral and basic amino acid transport protein rBAT and H/ACA ribonucleoprotein complex subunit 1 (Supplementary Table 5). Additional hierarchical clustering analysis showed that distinct expression patterns were displayed by VF and AFV groups of Asia II 7 and MEAM1 whiteflies. The expression profiles of all whitefly groups in response to CLCuMuV infection are shown in Supplementary Figure 3. Analysis of the biological variability of samples by PCA further confirmed the stability and reliability of data (Supplementary Figure 4). Additional analysis using qPCR showed that the observed transcriptional changes among both whitefly cryptic species were induced by the CLCuMuV infection (Supplementary Figure 5). The quantification of CLCuMuV-CP at 6 and 12 h AAP demonstrated that accumulation of the virus was significantly higher among Asia II 7 cryptic species as compared to that of MEAM1 whiteflies fed on the CLCuMuV-infected cotton plants (Figure 2).

**TABLE 2:** Comparative analysis of differentially expressed genes among whiteflies (Asia II 7 and MEAM1 cryptic species) fed for 6 and 12 h on CLCuMuV-infected or uninfected (0 h) cotton plants.

Comparative group	Differentially expressed genes of *Bemisia tabaci*		
	
	Total DEGs	Significantly up-regulated	Significantly down-regulated
CV6H vs CV0H	153	12	141
CV12H vs CV0H	141	3	138
CV12H vs CV6H	35	5	30
B6H vs B0H	445	251	194
B12H vs B0H	347	171	176
B12H vs B6H	52	13	39
CV0H vs B0H	8041	4660	3381
CV6H vs B6H	9235	4890	4345
CV12H vs B12H	8725	4854	3871

### Comparative gene expression tendencies among viruliferous and aviruliferous whiteflies

**FIGURE 3:**
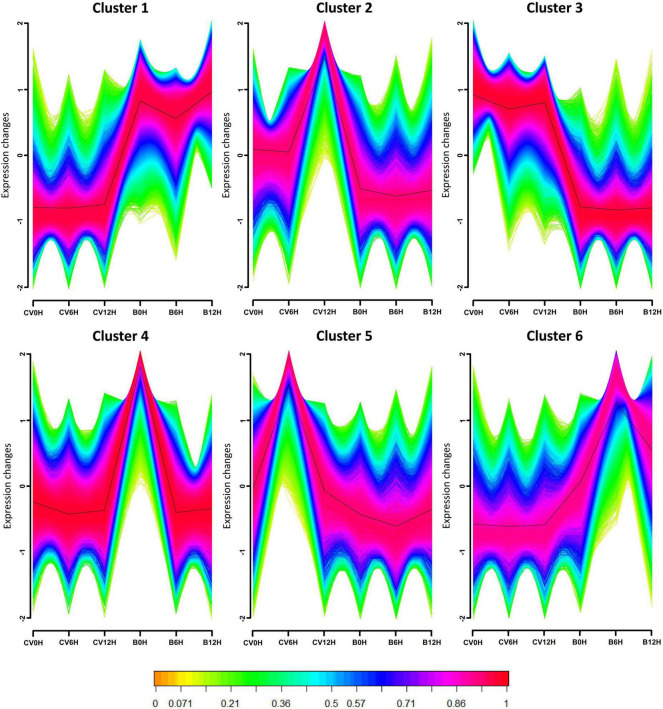
Time-based gene cluster analysis indicates the genes displaying alike expression patterns among different treatments (VF and AVF Asia II 7 and MEAM1 whiteflies) were grouped into different clusters. The horizontal axis denotes acquisition access time while the vertical axis represents the changes in gene expression. Normalized and transformed gene expression data were subjected to fuzzy c- means (FCM) clustering algorithm. A membership value in the range of 0-1 was used for clustering. Green and yellow lines denote the genes with a low membership value. The clustering cores composed of genes with membership value ≥0.90 are colored pink and red.

To further investigate the expression profiles of the assembled transcripts in response to CLCuMuV infection, we created the expression models of all DEGs, dividing VF and AVF groups of Asia II 7 and MEAM1 whiteflies into 6 unique clusters according to the log_2_ fold change (log_2_ FC) values (Figure 3). The results showed that most DEGs have variable patterns of expression in CLCuMuV-infected whiteflies as compared to uninfected ones. For instance, in clusters 1 and 6, most of the Asia II 7 genes had a lower expression as compared to those of MEAM1 after CLCuMuV infection. On the contrary, in clusters 2, 3, and 5, this expression pattern among Asia II 7 whiteflies was reversed, i.e., it remained higher than that of MEAM1 whiteflies. As for the expression patterns of DEGs grouped in cluster 4, VF Asia II 7 and MEAM1 did not show obvious variations except for the AVF MEAM1, where the expression was higher than that observed in AVF Asia II 7 whiteflies (Figure 3).

### Gene ontology and Kyoto encyclopedia of genes and genomes-based functional annotation of differentially expressed genes

**FIGURE 4:**
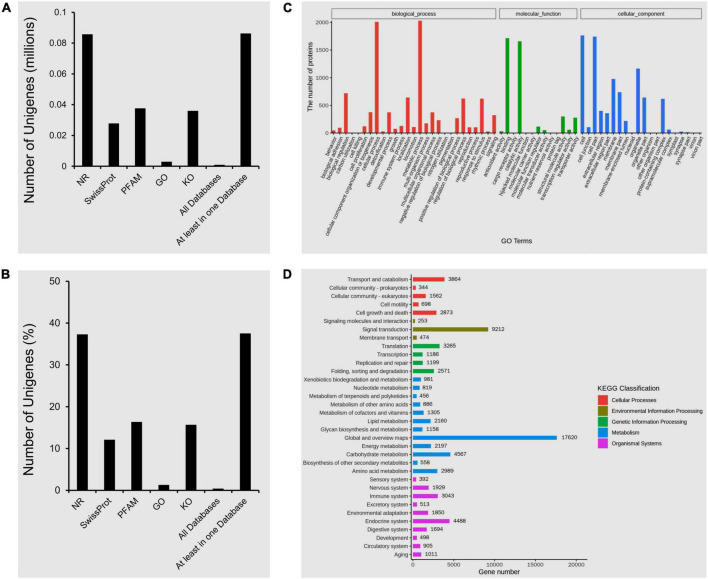
Functional annotation of DEGs. **(A)** The number of unigenes annotated in different databases; **(B)** percentage of unigenes annotated in NR, SwissProt, PFAM, GO and KO databases; **(C)** Gene ontology (GO) enrichment analysis of DEGs. GO analysis characterized the DEGs under biological process (BP), molecular function (MF) and cellular component (CC) categories; **(D)** KEGG analysis of DEGs from VF and AVF whiteflies represents the number of genes associated with five functional classes.

All DEGs were subjected to NR, SwissProt, PFAM, GO, and KEGG analyses (Figure 4A). The highest percentage (37.3%) of DEGs was annotated with NR (Figure 4B). GO and KEGG analyses were performed for functional classification of all DEGs from whiteflies. These DEGs from VF and AVF whiteflies were annotated with 60 terms under biological process (BP), cellular component (CC) and molecular function (MF) (Figure 4C). The DEGs classified under BP were mostly enriched in cellular and metabolic processes. While those under the functional category of MF were highly enriched in binding function and catalytic activity. Finally, the most abundantly enriched DEGs under CC category belonged to cell or cell part-related functions (Figure 4C). Further, results of the KEGG analysis divided all DEGs into 34 terms and 5 functional classes comprising cellular processes, environmental information processing, metabolism and organismal systems. Results revealed that 17,620 DEGs were associated with “global and overview maps” function under “metabolism” class. This was followed by a group of 9,212 DEGs related to the function of “signal transduction” under “environmental information processing” category (Figure 4D).

### Pairwise comparisons-based genome pathway enrichment analyses of differentially expressed genes

**FIGURE 5:**
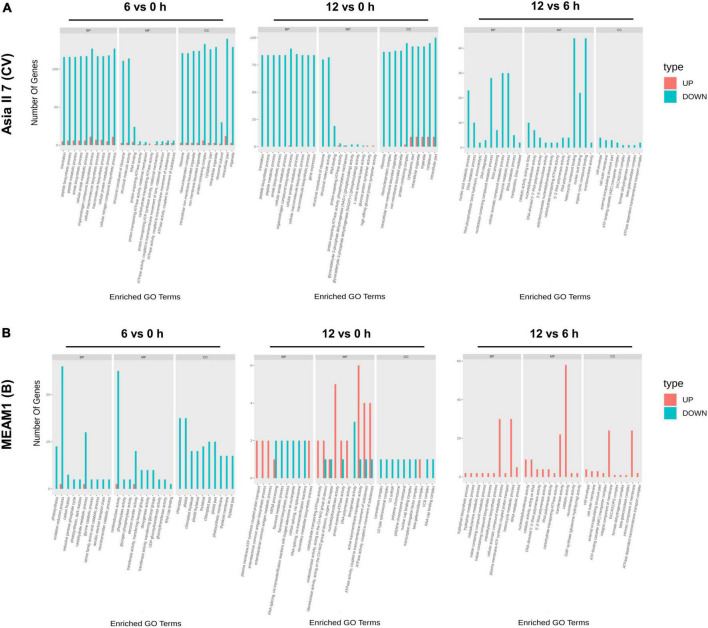
Gene ontology (GO) analysis represents the number of upregulated and downregulated genes enriched in different pathways among subgroups of **(A)** Asia II 7 and **(B)** MEAM1 whiteflies at 0, 6, and 12 h of CLCuMuV infection. The significance level based on the *p*-value is expressed by red (high) to yellow (low) colors.

To specifically determine the variability in the functional classification of DEGs, we performed GO enrichment analyses using pairwise comparisons among VF and AVF whiteflies at 6 and 12 h of CLCuMuV AAP (Figure 5). Results revealed that after 6 h of feeding on the CLCuMuV-infected plants, the most abundant group of downregulated DEGs under CC was enriched in intracellular part and protein-containing complex followed by cellular nitrogen and organonitrogen compound biosynthetic processes under BP. Likewise at 12 h time point, the downregulated DEGs in VF Asia II 7 were mostly enriched in CC-related pathways including cytoplasm, and intracellular part and protein-containing complex. The comparison among VF groups of Asia II 7 at 6 and 12 h time points showed that most of the downregulated genes were associated with organic cyclic and heterocyclic compound binding activities under MF (Figure 5A).

**FIGURE 6:**
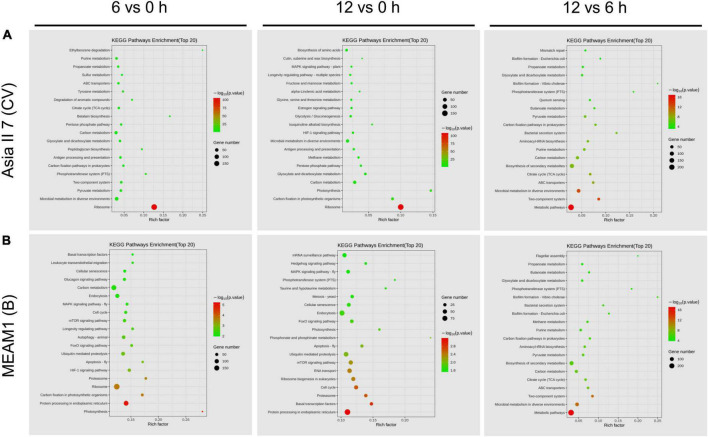
The Kyoto Encyclopedia of Genes and Genomes (KEGG) pathway enrichment plot among different comparative groups of viruliferous (VF) and aviruliferous (AVF) whiteflies [Asia II 7 **(A)** and MEAM1 **(B)**]. The vertical scale represents top 20 enriched pathways (path factor) while the horizontal scale represents the rich factor corresponding to the path factor. The dot color represents high (red) to low (green) changes in the size of *p*-value whereas the dot size represents the number of genes enriched in each pathway.

On the other hand, the downregulated DEGs of MEAM1 whiteflies at 6 h were mostly enriched in the oxidation-reduction process and oxidoreductase activity associated with BP and MF classes, respectively. In contrast, at 12 h, the highest number of upregulated DEGs among VF MEAM1 whiteflies was enriched in dioxygenase activity followed by nucleotidyltransferase activity under MF category. Finally, a comparison of VF MEAM1 at 6 and 12 h revealed that upregulated DEGs were mostly enriched in the transferase activity under MF category followed by heterocyclic metabolic and cellular aromatic compound metabolic activities under BP class (Figure 5B). Additionally, the comparison of VF Asia II 7 and MEAM1 showed that upregulated DEGs were mostly enriched in catalytic and hydrolase activities under MF class at 6 h however, DEGs related to both functional classes were downregulated among Asia II 7 at 12 h AAP (Supplementary Figure 6). In the KEGG database, the top 20 highly (*p* ≤ 0.05) enriched pathways were associated with “ribosome”, “metabolic pathways” and “protein processing in endoplasmic reticulum” among all pairwise comparisons (Figures 6A,B).

### Detection of the most abundant transcription factors among viruliferous Asia II 7 and MEAM1 whiteflies induced by CLCuMuV infection

**FIGURE 7:**
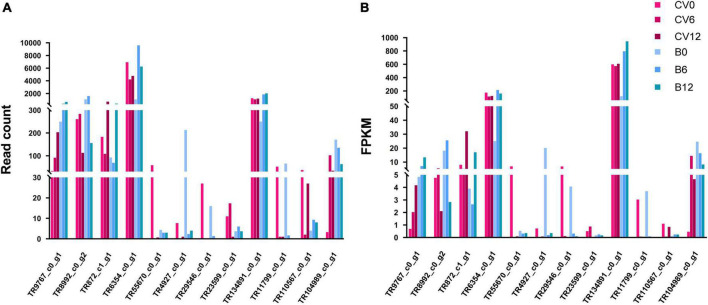
Changes in the **(A)** read counts and **(B)** expression levels of 12 highly responsive transcription factors at early stage of CLCuMuV infection of whiteflies (Asia II 7 and MEAM1).

We further investigated and compared the TFs-related variations among Asia II 7 and MEAM1 whiteflies. A total of 72 TF families were detected to have DEGs in both cryptic species following CLCuMuV infection (Supplementary Table 6). We found that most of the up- and down-regulated genes of VF Asia II 7 whiteflies were associated with four (HTH_1, HTH_AraC, SirB and zf-C2H2) families of TFs and notably, the highest number of DEGs were related to HTH_1 TF family (Table 3 and Supplementary Figure 7). On the contrary, CLCuMuV infection-induced TFs variability was lower in MEAM1 whiteflies i.e., the most abundant TFs among VF MEAM1 belonged to two families (zf-C2H2 and HTH_1). Also, the number of DEGs associated with zf-C2H2 TF family was dramatically increased among VF MEAM1 whiteflies (Table 3). We further selected the top 12 TFs that were up-regulated and down-regulated in response to CLCuMuV infection and analyzed the transcriptome data to compare differences in the read count and FPKM values associated with these TFs (Table 4 and Figure 7).

**TABLE 3:** The most abundantly detected TFs among VF whiteflies in response to CLCuMuV infection.

Comparative group	CLCuMuV-induced transcription factors		
	
	TF name	TF family	No. of genes
CV6H vs CV0H	HTH_1	bacterial regulatory helix-turn-helix protein, lysR family	14
CV12H vs CV0H	HTH_AraC	bacterial regulatory helix-turn-helix proteins, AraC family	1
	SirB	invasion gene expression up-regulator, SirB	1
	zf-C2H2	zinc finger, C2H2 type	1
	GerE	bacterial regulatory proteins, luxR family	1
CV12H vs CV6H	HTH_1	bacterial regulatory helix-turn-helix protein, lysR family	55
B6H vs B0H	zf-C2H2	zinc finger, C2H2 type	37
B12H vs B0H	zf-C2H2	zinc finger, C2H2 type	33
B12H vs B6H	HTH_1	bacterial regulatory helix-turn-helix protein, lysR family	53
CV6H vs B6H	zf-C2H2	zinc finger, C2H2 type	170
CV12H vs B12H	zf-C2H2	zinc finger, C2H2 type	161

**TABLE 4:** Important CLCuMuV-regulated candidate DEGs in Asia II 7 and MEAM1 whiteflies.

Cryptic species	Comparative group	Gene ID	Annotation	Read count change		Direction	FC	*p*-value (adj)
								
				from	to			
Asia II 7	CV6 vs. CV0	TR104989_c0_g1	LSM14-like protein B	3.23	99.24	Up	4.9466	0.000156
		TR55670_c0_g1	GPI-anchored protein 58	54.19	0	Down	−8.2269	1.09E-07
	CV12 vs. CV0	TR9767_c0_g1	alpha-glucosidase	35	190.51	Up	2.4436	4.11E-11
		TR29546_c0_g1	cuticle protein 8	27.7	0	Down	−7.3245	0.006906
	CV12 vs. CV6	TR110567_c0_g1	cytochrome P450 4C1	2.24	25.52	Up	3.5411	0.047502
		TR23599_c0_g1	phosphoenolpyruvate carboxykinase [GTP]	18.51	0.94	Down	−4.3432	0.006437
MEAM1	B6 vs. B0	TR6354_c0_g1	Mitochondrial phosphate carrier	1040.76	9398.82	Up	3.1753	4.65E-23
		TR4927_c0_g1	DNA-directed RNA polymerase II subunit RPB1	233.47	2.29	Down	−6.6705	1.20E-05
	B12 vs. B0	TR134891_c0_g1	myosin heavy chain	238.89	2083.31	Up	3.1268	1.10E-27
		TR11799_c0_g1	retinitis pigmentosa 1	62.61	0.35	Down	−7.3998	5.35E-07
	B12 vs. B6	TR872_c1_g1	cathepsin F-like protease	64.53	402.65	Up	2.6405	1.66E-15
		TR8992_c0_g2	heat shock protein 70	1473.67	161.64	Down	−3.189	1.10E-54

### Validation of candidate genes expressions by RT-qPCR

**FIGURE 8:**
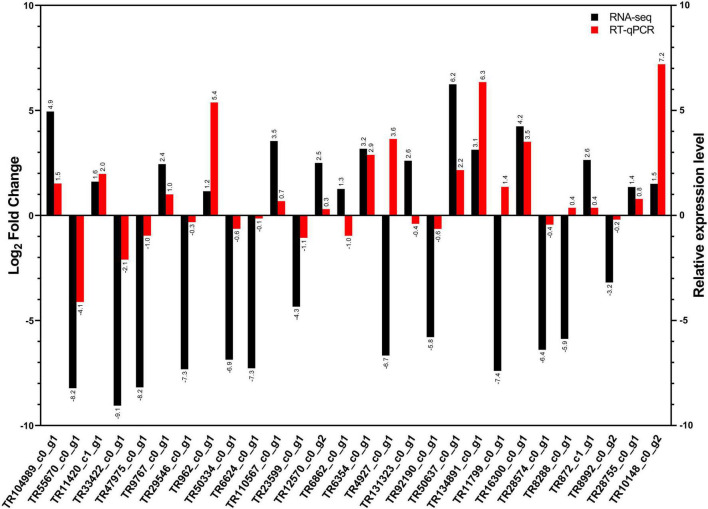
RNA-seq and RT-qPCR-based comparative expression analysis of 28 candidate genes of whiteflies (*Bemisia tabaci*, Asia II, and MEAM1) in response to CLCuMuV infection at 6 and 12 h. Each experiment was replicated with three independent biological replicates and three technical replicates were included per biological replicate.

We compared the mRNA expression profiles of 28 genes among VF and AVF groups of whiteflies using RT-qPCR. Of these, 23 genes exhibited a concordant change while 5 genes showed different expressions for both RT-qPCR and RNA-seq (Figure 8). Notably, the fold change intensity of the expression obtained by RT-qPCR was generally lower than that detected by RNA-seq (Figure 8), which might be attributed to the relatively higher sensitivity of the RNA-seq technique used in this experiment. Nonetheless, RT-qPCR results validated the directional changes obtained by DEG, implying the reliability of DEG results and that the DEGs identified in our study have high accuracy.

## Discussion

To date, several studies on begomovirus-whitefly interactions have been conducted providing valuable information on the transcriptional variations among different cryptic species of virus-infected whiteflies (Kaur et al., 2017; Geng et al., 2018; Hasegawa et al., 2018; Ding et al., 2019; Li et al., 2020; Nekkanti et al., 2022). However, the specific information on the CLCuMuV-whitefly interactions remains limited. Also, how an early CLCuMuV infection of different whitefly cryptic species affects the landscape of gene expression remains unknown. Here we performed transcriptomic profiling and compared the molecular responses of Asia II 7 and MEAM1 whitefly cryptic species after 6 and 12 h of CLCuMuV infection. A comparison of transcripts between VF and AVF groups of CLCuMuV-infected whiteflies revealed several genes that were highly responsive to the viral infection. Following 6 and 12 h of CLCuMuV acquisition, among Asia II 7 whiteflies, the most significantly expressed genes included LSm14-like protein B, GPI-anchored protein 58, alpha-glucosidase, cuticle protein 8, cytochrome P450 4C1 and phosphoenolpyruvate carboxykinase [GTP] while among MEAM1 whiteflies, mitochondrial phosphate carrier, DNA-directed RNA polymerase II subunit RPB1, myosin heavy chain, retinitis pigmentosa 1, cathepsin F-like protease and heat shock protein 70 were significantly (up or down) regulated. Further analyses demonstrate that these genes are involved in several pathways including galactose, biological transport, starch and sucrose metabolism, protein digestion and absorption, oxidoreductase activity, autophagy and apoptosis, hormone signaling, MAPK, cGMP-PKG signaling pathway and other important pathways associated with immune responses. Our findings reveal several candidate genes that might play vital roles in the regulation of complex begomovirus-whitefly interactions.

Remarkably, monitoring of the CLCuMuV-CP at 6 and 12 h post AAP demonstrated that Asia II 7 cryptic species were able to efficiently accumulate the virus compared with poor virus acquisition ability of the MEAM1 whiteflies (Figure 2). This reflects the competence of Asia II 7 whiteflies to efficiently acquire and subsequently transmit the virus. These findings are in line with results of a previous study documenting that Asia II 7, MED and MEAM1 cryptic species were able to acquire the virus as early as 6 h post feeding on the virus sources, however, only Asia II 7 whiteflies were able to efficiently transmit the virus (Chen et al., 2019). Previously, several studies have demonstrated that discrepancy in the vector-mediated transmission of begomoviruses essentially depends on the efficient virus acquisition and accumulation by a specific whitefly cryptic species/biotype.

The Sm-like (LSm) proteins from yeast have been associated with mRNA metabolism by promoting mRNA decapping and decay (Tharun et al., 2000). In the present study, we found that among Asia II 7 whiteflies, the expression of LSm14-like protein B was significantly up-regulated immediately following CLCuMuV acquisition, though its expression was higher among MEAM1 whiteflies. Previously, a study involving human cell lines has demonstrated that a processing body (P-body)-associated LSm14A protein acts as a sensor of the viral nucleic acids (both DNA and RNA) and plays a vital role in the activation of the cellular antiviral innate immunity via induction of IFN-β at an early stage of viral infection (Li et al., 2012). The targeted knock-down of LSm14A in mice resulted in the impairment of MITA/STING-mediated antiviral signaling pathway in a cell-specific manner and correlated with its regulatory role in initiating antiviral cytokines triggered by DNA viruses (Liu et al., 2016). In addition to LSm14, we also found phosphoenolpyruvate carboxykinase (PEPCK) that was significantly down-regulated among CLCuMuV-infected Asia II 7 whiteflies. Although this enzyme is known for metabolic activities associated with glyceroneogenic and gluconeogenic pathways (Ko et al., 2018), a previous study demonstrates that in pepper plants, the pepper phosphoenolpyruvate carboxykinase (CaPEPCK1) positively contributes to immunity against oomycete and bacterial pathogens (Choi et al., 2015). More recently, researchers have reported that BmPEPCK1 and BmPEPCK2 (isoforms of PEPCK) from *Bombyx mori* play a vital role in antiviral immunity by suppressing the multiplication of the nucleopolyhedrovirus (NPV) and enhancing the expression of autophagy-related genes (ATGs) (Guo et al., 2019). Nonetheless, the putative roles of LSm14 and GTP in begomovirus-whitefly interactions remain in the shadow.

Cathepsin proteases are the most abundant proteolytic enzymes present in the acidic lysosomal partitions where they function in energy metabolism, intracellular degradation of proteins and immune-related responses (Yadati et al., 2020). The functions of cathepsins in signaling, apoptosis and virus transmission have been well-documented (Kubo et al., 2012; Sim et al., 2012; Saikhedkar et al., 2015). In our study, a cathepsin protease (F) was highly up-regulated among MEAM1 whiteflies following CLCuMuV acquisition. This result is in agreement with previous findings where two cathepsins (B and D) were overexpressed in the silkworm cells infected with baculovirus (Gui et al., 2006; Wu et al., 2011). Similarly, MsCath1 and MsCath2 cathepsins together with CathL and B were highly expressed among *Manduca sexta* in response to parasitism by *Cotesia congregate* bracovirus (Serbielle et al., 2009). This indicates that in addition to their roles in the abiotic stress responses (Serbielle et al., 2009), cathepsins also respond to viral infection. Notably, another study highlights the implication of cathepsin B in the transmission of the aphid-borne potato leafroll virus (PLRV). In this research, the cathepsin B was observed to co-localize with virions at the cell membrane of PLRV-infected aphids. The higher activity of cathepsin B along with other proteases was indirectly associated with decreased PLRV transmission while its chemical inhibition restored the aphid’s ability to transmit the virus (Pinheiro et al., 2016). As mentioned earlier, we observed that MEAM1 cryptic species are incompetent vectors of CLCuMuV compared to Asia II 7. Thus, it appears plausible that overexpression of cathepsins (and other immunity-related genes) among MEAM1 results in the reduced viral accumulation that leads to failure in CLCuMuV transmission. To validate this hypothesis, further studies are imperative to see how differential regulation of cathepsins (and other genes associated with lysosomal pathway) affects the acquisition and/or transmission of the CLCuMuV.

Furthermore, our findings revealed that expression of the alpha-glucosidase-encoding gene was highly up-regulated in Asia II 7 whiteflies at 12 h of CLCuMuV AAP. While glucosidases (alpha and beta) are mainly known to hydrolyze carbohydrates, they also play vital roles in pathogen defense and other cellular functions (Bourne and Henrissat, 2001). Interestingly, a recent study has reported the up-regulation of an alpha-glucosidase (Bta11975) among ToCV-infected MED whiteflies and RNAi-mediated knockdown of Bta11975 was directly associated with reduced viral acquisition and transmission by whiteflies (Lu et al., 2021). Likewise, the begomoviral (TYLCV) infection of whiteflies was also observed to up-regulate the expression of a gene encoding alpha-glucosidase (Hasegawa et al., 2018). However on the contrary, the infection of whiteflies with TYLCV, ToCV and cucurbit yellow stunting disorder virus (CYSDV) was shown to down-regulate the alpha-glucosidase expression (Kaur et al., 2017) which implies that regulation of carbohydrate metabolism among whiteflies is differential in response to different plant viruses and it might play crucial function that affects the efficacy of whiteflies to acquire and transmit viruses. Additionally, comparison of VF Asia II 7 at 12 h AAP indicated significant downregulation of an alpha-glucosidase gene (Supplementary Table 5). We hypothesize that downregulation of alpha-glucosidase by Asia II 7 to shut down their carbohydrate metabolism might be associated with virus acquisition as observed for criniviruses (Kaur et al., 2019). Secondly, as demonstrated earlier, the suppression of alpha-glucosidase is related to compromised antiviral immune responses (Courageot et al., 2000; Chang et al., 2013; Bhushan et al., 2020), which could also be a reason behind the efficient virus acquisition by Asia II 7 cryptic species. Further experimental validation of these hypotheses might reveal intriguing findings highlighting the role of alpha-glucosidases in whitefly-mediated virus transmission.

The glycosylphosphatidylinositol (GPI) anchor represents a post-translational modification by which the modified proteins are anchored in the exterior leaflet of the cell membrane. The GPI-anchored proteins (GPI-Aps) possess structural and functional diversity and are associated with several biological processes (Paulick and Bertozzi, 2008; Heider et al., 2016). We identified a significantly down-regulated GPI-anchored protein 58 in Asia II 7 whiteflies at 6 h of viral AAP. The role of GPI-APs in viral pathogenesis and virus-host interactions have been extensively reviewed for the viruses that infect humans, mammals, birds and other animal species (Yu et al., 2019). Previously, the findings of a research revealed that the CP of pea enation mosaic virus (PEMV) interacts with a membrane alanyl aminopeptidase N (APN) with or without fusion with GPI. The authors concluded that APN is a functional receptor for the PEMV-CP in the gut of the pea aphid (*Acyrthosiphon pisum*) (Linz et al., 2015). Although GPI-APs together with myosin heavy chain have been identified in the lysosomes or endosome structures of begomoviral-infected whiteflies (Xia et al., 2018), their direct association in the inter-cellular trafficking or vector-borne transmission of begomoviruses is poorly understood. Future studies focused on the disruption of virus-receptor interactions might lead to the discovery and development of non-chemical strategies for the management of insect-transmitted plant viruses.

The scavenger class B receptor protein sequestosome-1 (SQSTM1) or p62 was also downregulated among VF Asia II 7 whiteflies at 12 h AAP. Reportedly, the upregulation of this autophagosome cargo protein in begomovirus-infected MEAM1 whiteflies was associated with virus resistance (Luan et al., 2011; Wang B. et al., 2016). Another study has demonstrated that SQSTM1 or p62 plays a vital role in the initiation of host antiviral immune responses. This receptor protein was associated with selective autophagy by inhibiting replication and targeting the Seneca Valley virus (SVV) to phagophores for degradation (Wen et al., 2021). Thus, it is reasonable to assume that downregulation of SQSTM1 or p62 in VF Asia II 7 might be a viral strategy to avoid whitefly-mediated antiviral defense responses. Additionally, we also found that the expression of H/ACA ribonucleoprotein complex subunit 1 and neutral and basic amino acid transport protein rBAT was highly accelerated in response to CLCuMuV infection at 12 h AAP. However, how these proteins are associated with whitefly-borne acquisition and/or transmission of begomoviruses remains elusive.

Vitellogenin (Vg) is another insect protein that has been extensively studied in plant-virus interactions. We found that the expression of Vg was significantly suppressed among Asia II 7 while it was accelerated among MEAM1 whiteflies at 12 h AAP. Researchers have demonstrated that tissue-specific processing of Vg from *Laodelphax striatellus* mediates the transmission of rice stripe virus (RSV) (Huo et al., 2018). Likewise, RSV was shown to hitchhike the Vg ligand receptor pathways of its insect vector (*L. striatellus*) to attain cell entry and subsequent vertical transmission (Huo et al., 2019). Remarkably, the role of whitefly Vg in the transovarial transmission of TYLCV has been linked with evolution and global spread of begomoviruses (Wei et al., 2017). Recently, it was found that a midgut-synthesized Vg plays functional role during whitefly-mediated transmission by TYLCV by facilitating the movement of virions across the midgut barriers of whitefly vector (He et al., 2021). Despite the well-known role of insect Vgs in the virus transmission, it is yet unknown how CLCuMuV manipulate this receptor protein to attain differential transmission by whitefly cryptic species.

Notably, we also found a cuticle protein 8 with significantly low expression following CLCuMuV infection of Asia II 7 whiteflies. Hitherto, cuticular proteins (CuPs) have been designated as the emerging vector molecules that are known to regulate the transmission of phytoviruses, regardless of the mode of their insect-mediated transmission (Deshoux et al., 2018). While in the case of non-circulative transmission of phytoviruses CuPs are the obvious candidate receptors (Webster et al., 2017; Bahrami Kamangar et al., 2019; Deshoux et al., 2020), they are well known to interact with viruses during circulative transmission by facilitating virion entry into the gut and the hemolymph (Deshoux et al., 2018). For instance, it has been demonstrated that CPR1, a cuticular protein of *Laodelphax striatellus* (small brown planthopper) interacts with pc3 (nucleocapsid protein) of the rice stripe virus (RSV) and facilitates viral survival in the hemolymph (Liu et al., 2015). Likewise, for the viruses transmitted in a circulative manner, several CuPs have been reported to play vital roles in the virus-insect interactions including N and CPR-2 of aphids infected with poleroviruses and luteoviruses (Seddas et al., 2004; Cilia et al., 2011; Wang et al., 2015), respectively and cuticle protein A3A-like of planthoppers infected with tenuiviruses (Li et al., 2018). Our findings are consistent with previous reports describing the down-regulation of CuPs transcripts in different insect vectors infected with RSV, tomato spotted wilt virus (TSWV) and Southern rice black-streaked dwarf virus (SRBSDV) (Wang L. et al., 2016; Schneweis et al., 2017; Yang et al., 2018). Given that CuPs are integral membrane constituents, their reduced expression implies a slow-down developmental process to promote virus acquisition (Widana Gamage et al., 2018).

## Conclusion

We compared the transcriptional variations among *B*. *tabaci* (Asia II 7 and MEAM1 cryptic species) in response to the early infection of CLCuMuV. Our comparative transcriptomic profiling showed that the most abundant groups of DEGs among Asia II 7 and MEAM1 belonged to HTH1 and zf-C2H2 TFs families, respectively. Several genes were highly responsive to CLCuMuV infection including those associated with immune responses, biological transport, protein degradation and absorption, metabolic pathways and cellular receptors. Overall, the transcriptional changes and immune-related responses among MEAM1 whiteflies post-CLCuMuV acquisition were higher than that of Asia II 7. Our findings extend the standing knowledge of begomovirus-whitefly interactions by revealing potential molecular targets of CLCuMuV that might play vital roles in regulating the differential transmission of begomoviruses by different whitefly cryptic species. Further mechanistic investigations focusing on these candidate genes could reveal novel targets for the sustainable management of whitefly-transmitted phytoviruses.

## Data availability statement

The datasets presented in this study can be found in online repositories. The names of the repository/repositories and accession number(s) can be found below: NCBI, PRJNA865034.

## Author contributions

TF and QL performed the experiments, acquired, and analyzed the data. TF, QL, XS, TC, and YT wrote the manuscript. ZH and YT conceived and designed the experiments. All authors contributed to the article, read, and approved the submitted version.
