# Supplementary material for: Comparative transcriptome profiling reveals a network of differentially expressed genes in Asia II 7 and MEAM1 whitefly cryptic species in response to early infection of Cotton leaf curl Multan virus
Source: Front Microbiol. 2022 Oct 4;13:1004513. doi: 10.3389/fmicb.2022.1004513 (PMC9577181; doi:10.3389/fmicb.2022.1004513)
Supplement: Supplementary file 1 [file Data_Sheet_1.docx]

Comparative transcriptome profiling reveals a network of differentially expressed genes in Asia II 7 and MEAM1 whitefly cryptic species in response to early infection of Cotton leaf curl Multan virus

Supplementary Material


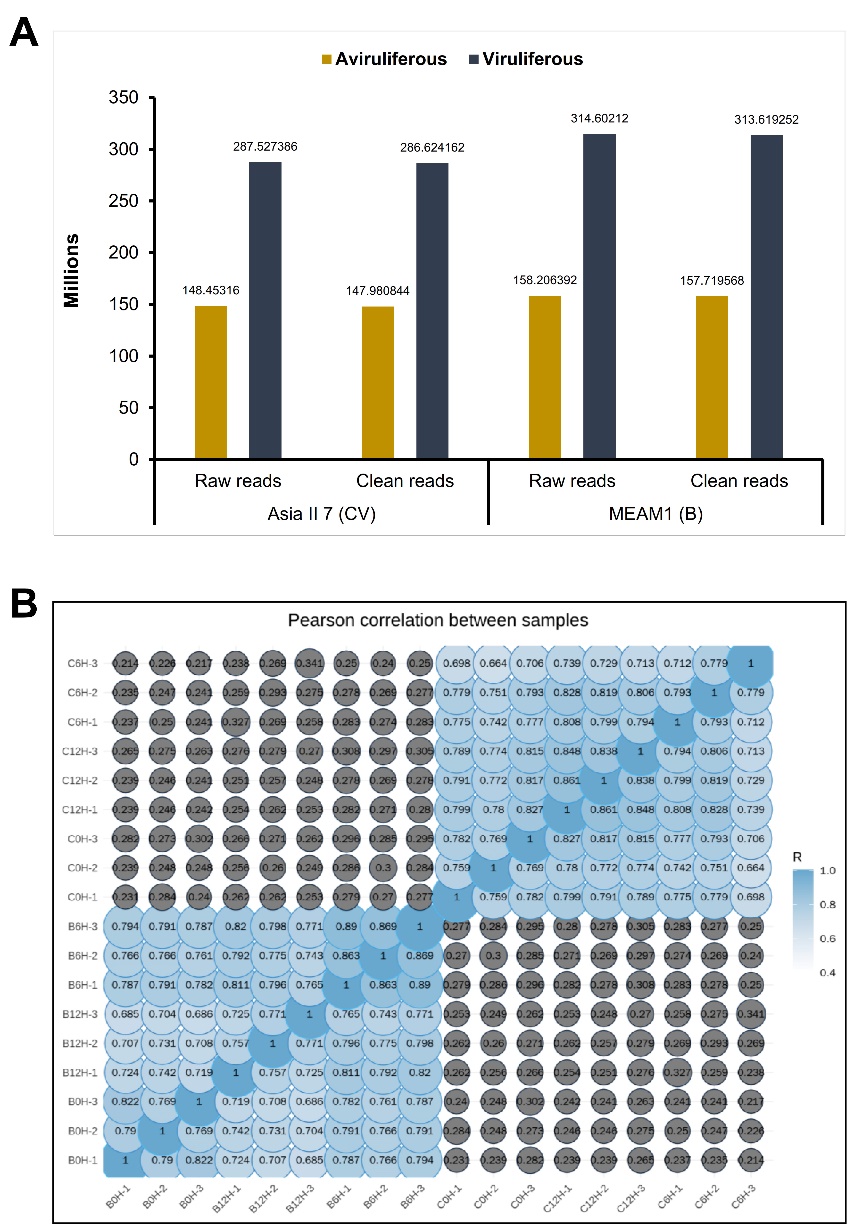


**Supplementary Figure S1.** Transcriptome analysis of viruliferous (VF) and aviruliferous (AVF) whiteflies. (A) Comparison of raw and clean reads obtained after RNA-seq of Asia II 7 and MEAM1 whitefly cryptic species fed on cotton leaf curl Multan virus (CLCuMuV) infected or healthy cotton plants for periods of 6 and 12 hours; (B) Pearson’s correlation matrix for replicate biological samples prepared from VF and AVF Asia II 7 and MEAM1 whiteflies fed on CLCuMuV-infected or healthy cotton plants for periods of 6 and 12 hours.


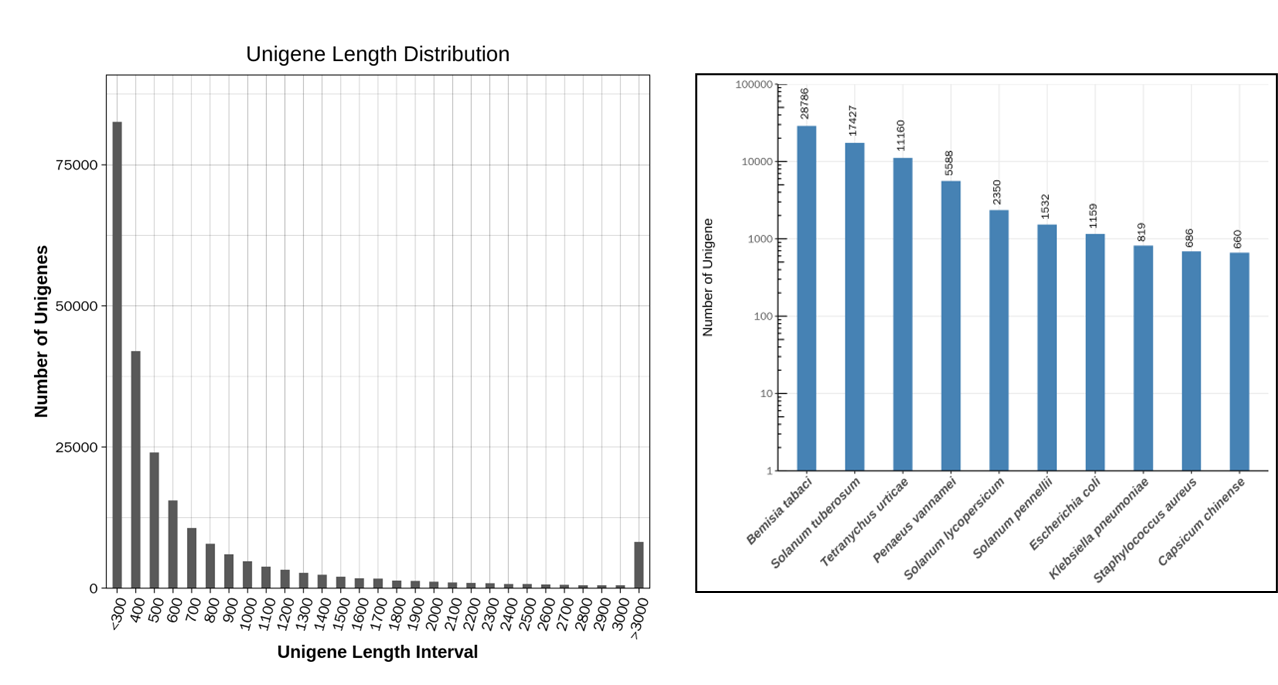


**Supplementary Figure S2.** Distribution of the unigene transcripts length (left) and NR-based comparison of mapped species (Right).


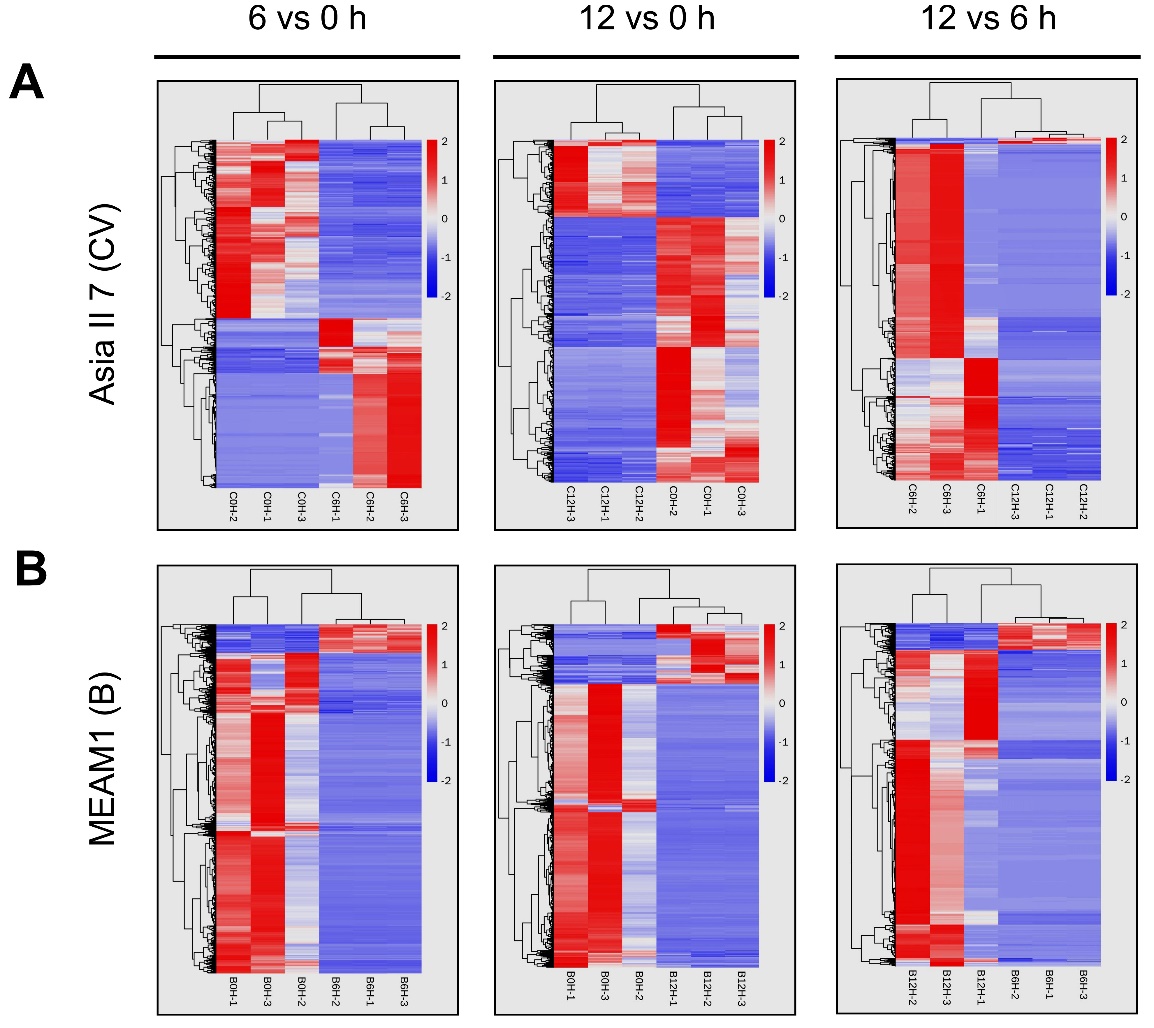


**Supplementary Figure S3.** Cluster heatmap depicting patterns of gene expression across all samples derived from VF and AFV whiteflies. Genes exhibiting alike expression patterns are clustered together. Red and blue colors represent high and low scales of relative gene expressions, respectively. Each gene is represented by a row while each replicate (sample) is represented by a column.


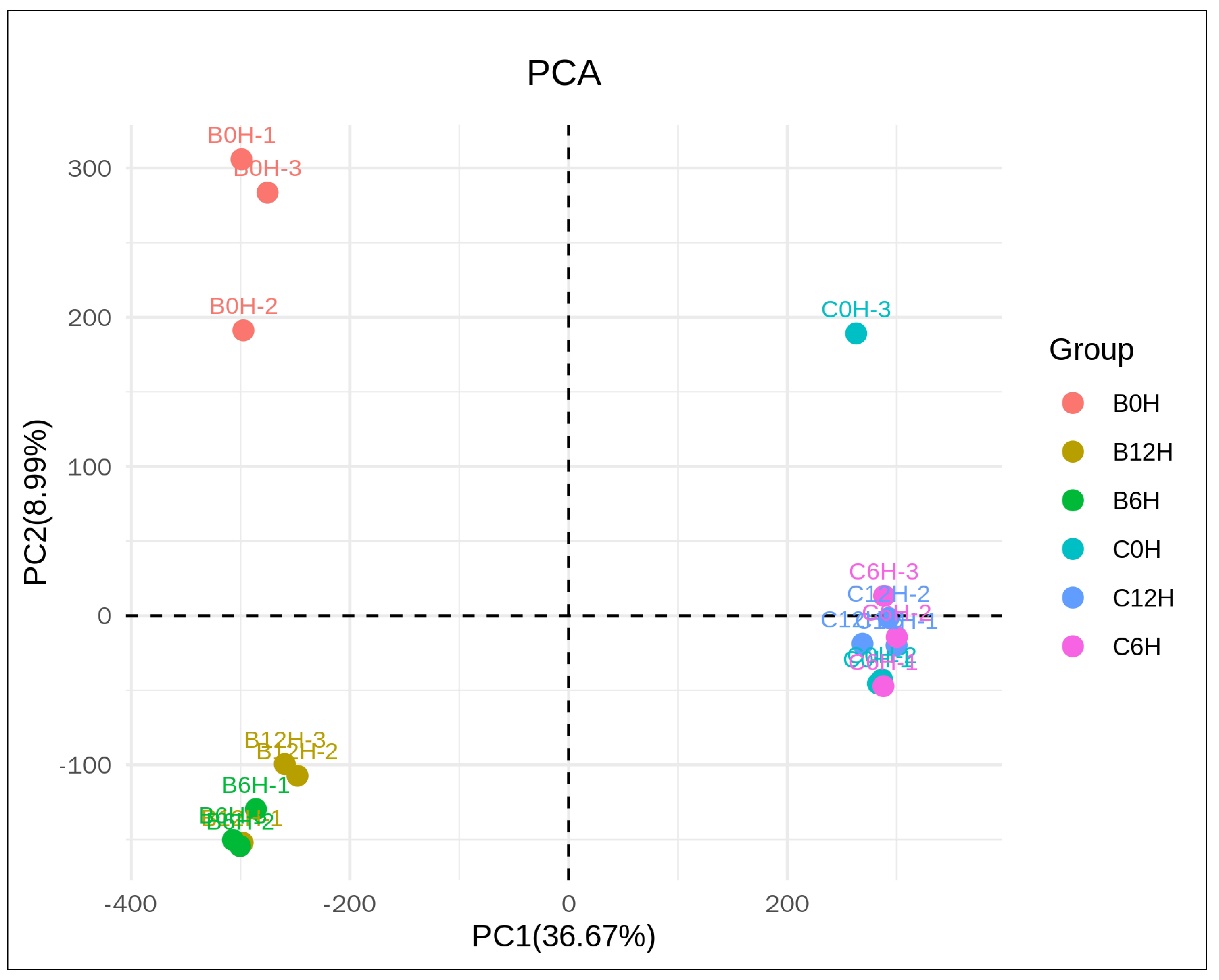


**Supplementary Figure S4.** Principal component analysis (PCA) plot was generated for 18 samples originated from viruliferous (VF) and aviruliferous (AVF) whiteflies (Asia II 7 and MEAM1) fed on healthy and CLCuMuV-infected plants.


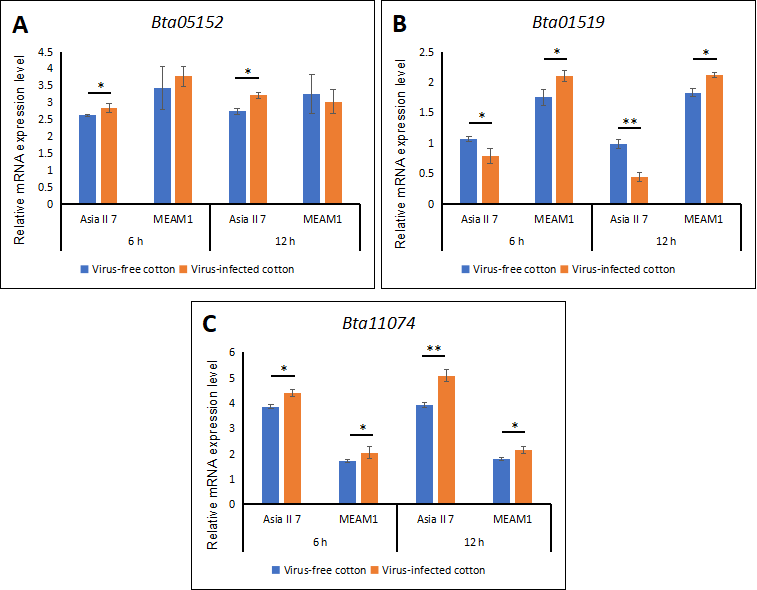


**Supplementary Figure S5.** Comparative gene expression analysis among Asia II 7 and MEAM1 cryptic species. (A-C) The transcriptional analysis of three genes (*Bta05152, Bta01519* and *Bta11074*) was performed using RNA of whiteflies fed on the virus-free and virus-infected *Gossypium hirsutum* cv. Xinhai plants for 6 and 12 hours. Student *t*-test was used to analyze the statistically significant differences between viruliferous and aviruliferous whitefly groups. Asterisks denote statistical significance: *, *P* < 0.05; **, *P* < 0.01.


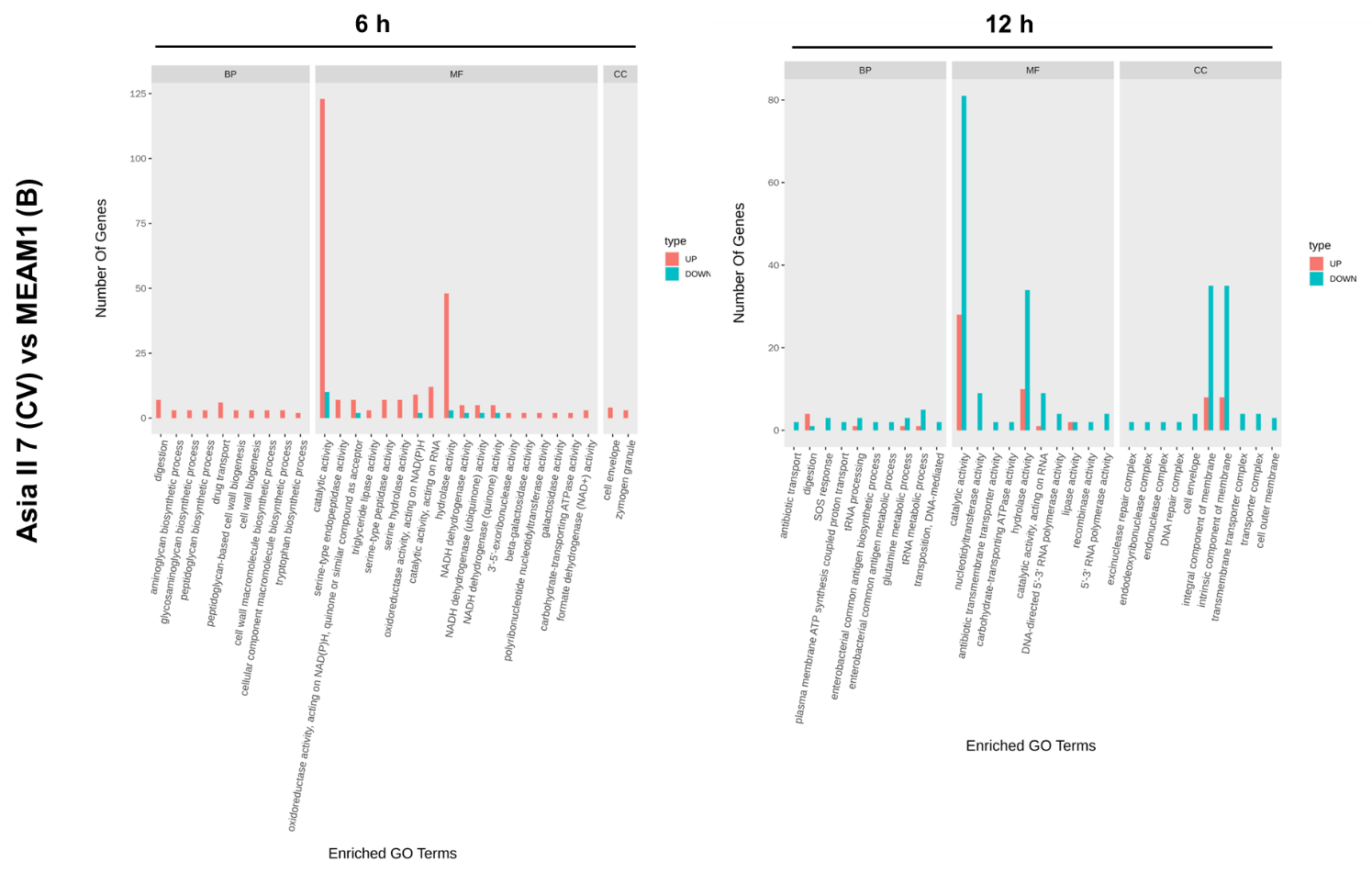


**Supplementary Figure S6.** Comparative Gene Ontology (GO) analysis of upregulated and downregulated DEGs between CLCuMuV-infected Asia II 7 and MEAM1 cryptic species at 6 and 12 h AAPs.


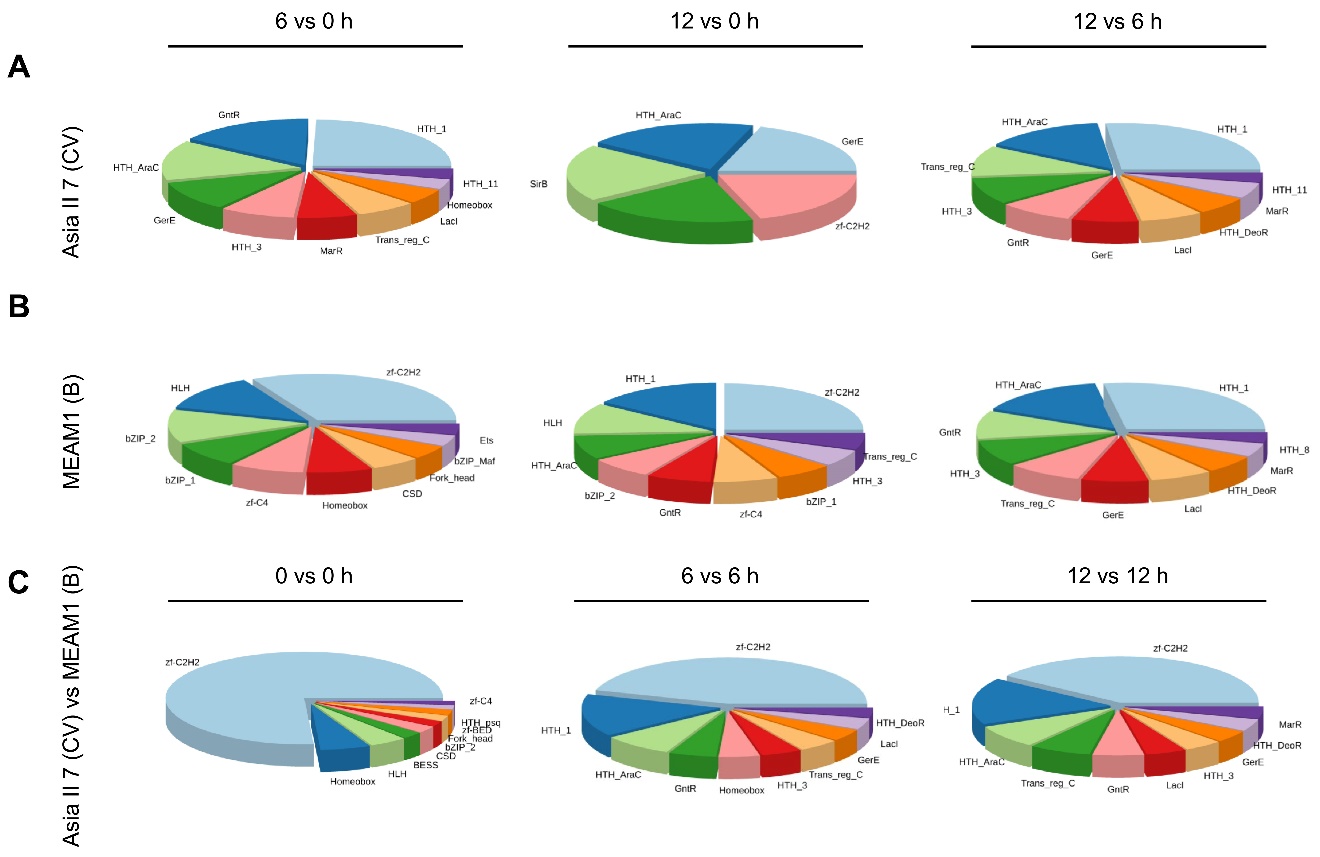


**Supplementary Figure S7.** Comparison of different transcription factor groups among Asia II 7 and MEAM1 whiteflies induced by CLCuMuV infection.

**Supplementary Table S1:** Details of primers used in this study.

| **Primer** | **Sequence (5'-3')** | **Target gene** | **Product size (bp)** | **Purpose** | **Reference** |
| --- | --- | --- | --- | --- | --- |
| mtCOI-C1- J- 2195 | TTGATTTTTTGGTCATCCAGAAGT | mitochondrial cytochrome oxidase subunit 1 | 880 | biotype verification | [1] |
| mtCOI-TL2N- 3014 | TCCAATGCACTAATCTGCCATATTA |  |  |  |  |
| CLCuMuV-CL-F | CAGGAAGCAGGAAAATACGAGA | coat protein, AC1, AC2 & AC3 | 831 | CLCuMuV detection | [2] |
| CLCuMuV-CL-R | TGGCAGTCCAACACAAAATACG |  |  |  |  |
| CLCuMuB-beta-F | AAGTCGAATGGAACGTGAATGT | βC1 | 837 | CLCuMuV detection | [2] |
| CLCuMuB-beta-R | GGAGACCAAAAGAGGAGAGAGA |  |  |  |  |
| CLCuMuV-CP-F | GGCTTTGGTCAAGAAGTTTGTC | coat protein | 120 | qPCR | present study |
| CLCuMuV-CP-R | GCGTGGGTACAAGCCATATAA |  |  |  |  |
| Bta02984-F | CCCAACCTCAGATCTACGACTA | LSM14-like protein B | 120 | qPCR | present study |
| Bta02984-R | GGAGCGGAGACTTGAACAATAG |  |  |  |  |
| Bta05743-F | CTGGTTGCCTACGCTGAA | ACYPI009429 protein | 120 | qPCR | present study |
| Bta05743-R | GGCCTCCATCGTCTTGTAG |  |  |  |  |
| Bta04719-F | TTAGCTGCTGATGTCGGTAAAT | mitochondrial ADP/ATP carrier protein | 120 | qPCR | present study |
| Bta04719-R | CTCCGAAACCTCTGTACAATCC |  |  |  |  |
| Bta11985-F | GCCTACCCTCACGCCTA | dihydrolipoyllysine-residue acetyltransferase | 120 | qPCR | present study |
| Bta11985-R | AATGGGTGAGCTCGGAGTA |  |  |  |  |
| Bta04612-F | AGTGCTTTCAGCCTCCATAAC | Unknown | 120 | qPCR | present study |
| Bta04612-R | ACGAGCTCGGTCTCTCC |  |  |  |  |
| Bta14422-F | GAGGGCACTCTCAACTCTTATG | alpha-glucosidase | 120 | qPCR | present study |
| Bta14422-R | GGTTCTGTTCCAAGGTTCACTA |  |  |  |  |
| Bta09571-F | CCTCTGAGCCAATCAAGTACC | cuticle protein 19 | 120 | qPCR | present study |
| Bta09571-R | TAATGTCCGTCACCGCTTTC |  |  |  |  |
| Bta14750-F | TGAAGGTTCTGCTAAGGAACTG | cathepsin B | 120 | qPCR | present study |
| Bta14750-R | AGGCTCCCTGGTTTGTAATG |  |  |  |  |
| Bta05142-F | GTGGACGAGGAAGACCATTT | uncharacterized protein LOC109037912 | 120 | qPCR | present study |
| Bta05142-R | GGGAAATCGCAGACAATGAAC |  |  |  |  |
| Bta12955-F | GTAGCTATCGTCCTGGCTTTG | cuticle protein 16.4 | 120 | qPCR | present study |
| Bta12955-R | GTGCGGAGGATGTTGGAG |  |  |  |  |
| Bta02661-F | TTCATCGGAGAGGGCAAATAC | cytochrome P450 | 120 | qPCR | present study |
| Bta02661-R | GTCCTCTACTGGTCAACAATCC |  |  |  |  |
| Bta09047-F | GTCGACGAGGTCCATCAATATC | Phosphoenolpyruvate carboxykinase [GTP] | 120 | qPCR | present study |
| Bta09047-R | CCGACAATCGCTCCCTTAAT |  |  |  |  |
| Bta10766-F | CTATGGAAGGCTGGCAAGAA | cathepsin B | 120 | qPCR | present study |
| Bta10766-R | CCGTCTTCAACATGGGTAGTG |  |  |  |  |
| Bta07851-F | GATTCAAGCTGCCAACCAATAC | vitellogenin | 120 | qPCR | present study |
| Bta07851-R | GCGAAGTCTTCTGGGTTCTT |  |  |  |  |
| Bta09696 -F | CAATCTGATCGGAGAGGAGAAC | alpha-glucosidase | 120 | qPCR | present study |
| Bta09696 -R | CAGATTCCATAGGGCTGAGAAG |  |  |  |  |
| Bta14060-F | GGCCTTCTTCAAATGGTTCATC | Mitochondrial phosphate carrier protein | 120 | qPCR | present study |
| Bta14060-R | CGGAAGATCCTTGTCCGTATC |  |  |  |  |
| Bta09555-F | GAAGGCGAGGTCACGTATTT | Unknown | 120 | qPCR | present study |
| Bta09555-R | TACTTCTCAAGTCAGCGGTAAAG |  |  |  |  |
| Bta00134-F | CAATCTGATCGGAGAGGAGAAC | Elongation factor 2 | 120 | qPCR | present study |
| Bta00134-R | CAGATTCCATAGGGCTGAGAAG |  |  |  |  |
| Bta07871-F | GAATCTGCTTCCCTCCGAAATA | Cholesterol desaturase daf-36 | 120 | qPCR | present study |
| Bta07871-R | GGGCAATAGGCATCAACAATG |  |  |  |  |
| Bta03792-F | GGGAAGAAAGCTGCAGAAGA | Ribosome-binding factor A | 120 | qPCR | present study |
| Bta03792-R | CATAATAGCGCCCTGATCCAA |  |  |  |  |
| Bta04967-F | GACAAGGTCAACCACCTTACA | Myosin-9, putative | 120 | qPCR | present study |
| Bta04967-R | TTCAACGTCTCCTCTCAGTTTC |  |  |  |  |
| Bta09171-F | AGCTGACGAAGAGGCAAATAA | Unknown | 120 | qPCR | present study |
| Bta09171-R | CTCCACATCACCACCAACTAC |  |  |  |  |
| Bta01005-F | GAAAGCCATCAAGGAGGAAGA | Unknown | 120 | qPCR | present study |
| Bta01005-R | CAGCTCTCTGTGACTTGTGAA |  |  |  |  |
| Bta09563-F | GCCTACAAATTCGCCTACGA | Cuticle protein | 120 | qPCR | present study |
| Bta09563-R | ACGAGAGAGTAAGATCCCTTGA |  |  |  |  |
| Bta09556-F | GGATACAAATCCTCCGGACAC | Unknown | 120 | qPCR | present study |
| Bta09556-R | GTACTTGTGGCTGGATTCCTTA |  |  |  |  |
| Bta05911-F | CACAGTGCAGGCTTCTTCTAA | Cathepsin F-like protease | 120 | qPCR | present study |
| Bta05911-R | CAACACAGGTCGAGAACGTATAG |  |  |  |  |
| Bta02903-F | AACCGCTGGTGGTGTTATG | Heat shock protein 70 | 120 | qPCR | present study |
| Bta02903-R | GCAGGTTGATTGTCGGAGTAG |  |  |  |  |
| Bta14126-F | GAGTCCGGTGACGTTCG | uncharacterized protein LOC109034817 | 120 | qPCR | present study |
| Bta14126-R | GCGGTGCTTGAGGATGTA |  |  |  |  |
| Bta14011-F | ACGCACAGACCGTATATGAATG | 2-oxo-4-hydroxy-4-carboxy-5-ureidoimidazoline decarboxylase | 120 | qPCR | present study |
| Bta14011-R | CGTGAGATCAGGGTACAAGTTTAG |  |  |  |  |
| Bta14116-F | GAACTTCGCGCCTATCATCA | Unknown | 120 | qPCR | present study |
| Bta14116-R | TCCCGAAGTAGTTGATCTCGTA |  |  |  |  |

**Supplementary Table S2:** Results of the qualitative and quantitative analyses of 18 RNA samples used in this study.

| **Sample name** | **OD (260/280)** | **OD (260/280)** | **Concentration (ng/ul)** | **RIN** |
| --- | --- | --- | --- | --- |
| CV0H-1 | 1.89 | 1.89 | 1350 | 8.9 |
| CV0H-2 | 1.92 | 1.93 | 1720 | 9.0 |
| CV0H-3 | 1.88 | 1.88 | 1620 | 9.1 |
| CV6H-1 | 1.92 | 1.89 | 1270 | 9.4 |
| CV6H-2 | 1.89 | 1.89 | 1790 | 9.3 |
| CV6H-3 | 1.88 | 1.88 | 1710 | 9.6 |
| CV12H-1 | 1.91 | 1.95 | 2470 | 9.2 |
| CV12H-2 | 1.95 | 1.89 | 922 | 9.3 |
| CV12H-3 | 1.93 | 1.93 | 1170 | 9.0 |
| B0H-1 | 1.89 | 1.88 | 1830 | 9.0 |
| B0H-2 | 1.89 | 1.92 | 1230 | 8.6 |
| B0H-3 | 1.88 | 1.86 | 1520 | 8.7 |
| B6H-1 | 1.89 | 1.91 | 983 | 8.8 |
| B6H-2 | 1.91 | 1.87 | 1160 | 9.0 |
| B6H-3 | 1.87 | 1.93 | 1300 | 9.0 |
| B12H-1 | 1.94 | 1.92 | 1280 | 9.1 |
| B12H-2 | 1.92 | 1.87 | 2410 | 8.5 |
| B12H-3 | 1.87 | 1.86 | 1280 | 8.5 |

**Supplementary Table S3:** Characteristics of different databases, methods and parameters used for gene functional annotation and pathway enrichment analyses.

| **Database** | **Description** | **Softwares, parameters and e-value thresholds** |
| --- | --- | --- |
| Nr | The non-redundant protein database in NCBI includes Swiss prot, PIR (protein information resource), PRF (Protein Research Foundation), PDB (Protein Data Bank) protein database and protein coding sequences of GenBank and RefSeq. | NCBI blast 2.6.0+  e-value= 1e-5 |
| Swiss-Prot | The database maintained by EBI (European Institute of Bioinformatics) contains the protein annotation information database with relevant references and collated by experienced biologists. | NCBI blast 2.6.0+  e-value= 1e-5 |
| Pfam | Pfam database establishes HMM statistical model of amino acid sequence of each family through protein sequence alignment, which is the most comprehensive classification system of protein domain annotation. | HMMER3.0  e-value=0.01 |
| GO | Go (gene ontology) database is an international standardized gene function classification system, which is used to comprehensively describe the functional attributes of gene coding products in organisms. It is mainly divided into three categories: biological process, molecular function and cellular component, which respectively describe the biological process, molecular function and cellular environment in which the products encoded by genes participate. | Blast2GOv2.5 e-value=1e-6 |
| KEGG | KEGG (Kyoto Encyclopedia of Genes and Genomes) systematically analyzes the metabolic pathways of gene products and compounds in cells and the functions of these gene products. It integrates the data of genome, chemical molecules and biochemical systems, including metabolic pathway, drug, disease, genes and genome. | KAAS e-value=1e-10 |

**Supplementary Table S4:** Mapping results of clean reads from 18 samples associated with CLCuMuV-infected and uninfected whiteflies.

| **Sample name** | **Total reads** | **Total mapped** | **Total mapped (%)** | **GC (%)** |
| --- | --- | --- | --- | --- |
| B0H-1 | 52366826 | 43059316 | 82.23 | 41.85 |
| B0H-2 | 62397588 | 50815820 | 81.44 | 42.24 |
| B0H-3 | 42955154 | 35845330 | 83.45 | 42.47 |
| B12H-1 | 54801270 | 45192180 | 82.47 | 42.13 |
| B12H-2 | 45232384 | 36837582 | 81.44 | 41.58 |
| B12H-3 | 47747744 | 39242012 | 82.19 | 43.07 |
| B6H-1 | 49866964 | 41434458 | 83.09 | 40.36 |
| B6H-2 | 61026362 | 50686898 | 83.06 | 39.46 |
| B6H-3 | 54944528 | 45847784 | 83.44 | 41.32 |
| CV0H-1 | 51248146 | 41640116 | 81.25 | 43.6 |
| CV0H-2 | 50174214 | 41005020 | 81.73 | 41.08 |
| CV0H-3 | 46558484 | 38463304 | 82.61 | 43.05 |
| CV12H-1 | 48529992 | 40008510 | 82.44 | 43.02 |
| CV12H-2 | 43020410 | 35140292 | 81.68 | 42.74 |
| CV12H-3 | 58605038 | 47323320 | 80.75 | 40.5 |
| CV6H-1 | 50668854 | 41632726 | 82.17 | 42.86 |
| CV6H-2 | 44746026 | 36717470 | 82.06 | 43.51 |
| CV6H-3 | 41053842 | 33205700 | 80.88 | 44.49 |

**Supplementary Table S5:** Comparative analysis of significantly down- and up-regulated DEGs among VF Asia II 7 and MEAM1 cryptic species after 12 h AAP.

| **Transcript ID** | **Gene ID** | **Function** | **Comparative group** | **Direction** | **Log_2_ FC value** |
| --- | --- | --- | --- | --- | --- |
| TR23599_c0_g1 | *Bta09047* | Phosphoenolpyruvate carboxykinase [GTP] | CV12 vs CV6 | down | -4.3432 |
| TR59722_c0_g1 | *Bta09696* | Alpha-glucosidase | CV12 vs CV6 | down | -2.3654 |
| TR39451_c0_g1 | *Bta04389* | Sequestosome-1 isoform X1 | CV12 vs CV6 | down | -1.7267 |
| TR10703_c0_g1 | *Bta01618* | Protein msta, isoform A | CV12 vs CV6 | down | -1.676 |
| TR90536_c0_g1 | *Bta07852* | Vitellogenin | CV12 vs CV6 | down | -1.6644 |
| TR872_c1_g1 | *Bta05911* | Cathepsin F-like protease | B12 vs B6 | up | 2.6405 |
| TR6862_c0_g1 | *Bta07851* | Vitellogenin | B12 vs B6 | up | 1.5161 |
| TR10148_c0_g2 | *Bta14126* | Uncharacterized protein | B12 vs B6 | up | 1.5068 |
| TR10172_c0_g1 | *Bta07377* | Neutral and basic amino acid transport protein rBAT | B12 vs B6 | up | 1.3615 |
| TR28755_c0_g1 | *Bta14116* | Probable H/ACA ribonucleoprotein complex subunit 1 | B12 vs B6 | up | 1.3555 |

**Supplementary Table S6:** TF families detected among Asia II 7 and MEAM1 in response to CLCuMuV infection.

| **TF families detected among Asia II 7 and MEAM1 in response to CLCuMuV infection** | | | | | | **All TF families** |
| --- | --- | --- | --- | --- | --- | --- |
| **B6 vs B0** | **B12 vs B0** | **B12 vs B6** | **CV6 vs CV0** | **CV12 vs CV0** | **CV12 vs CV6** |  |
| zf-C2H2 | AF-4 | AraC_N | AraC_N | HTH_AraC | HTH_3 | AF-4 |
| zf-C4 | AP2 | Arg_repressor | Crp | zf-C2H2 | AraC_N | AP2 |
| zf-C5HC2 | AraC_N | bZIP_1 | FlhC | GerE | Arg_repressor | AraC_N |
| zf-MIZ | bZIP_1 | bZIP_2 | FlhD | SirB | CoiA | Arg_repressor |
| zf-TAZ | bZIP_2 | Crl | GerE |  | Crl | bZIP_1 |
| Zim | bZIP_Maf | Crp | GntR |  | Crp | bZIP_2 |
|  | CBFB_NFYA | Fe_dep_repress | Homeobox |  | DUF24 | bZIP_Maf |
|  | CP2 | FlhC | HTH_1 |  | Fe_dep_repress | CBFB_NFYA |
|  | Crp | FlhD | HTH_11 |  | FlhC | CoiA |
|  | CSD | FUR | HTH_3 |  | FlhD | CP2 |
|  | DM | GerE | HTH_5 |  | FUR | Crl |
|  | E2F_TDP | GntR | HTH_6 |  | GerE | Crp |
|  | EIN3 | HTH_1 | HTH_8 |  | GntR | CSD |
|  | Ets | HTH_11 | HTH_AraC |  | HTH_1 | DM |
|  | Fez1 | HTH_3 | HTH_DeoR |  | HTH_11 | DUF24 |
|  | FlhC | HTH_5 | KilA-N |  | HTH_3 | E2F_TDP |
|  | FlhD | HTH_6 | LacI |  | HTH_5 | EIN3 |
|  | Fork_head | HTH_8 | LexA_DNA_bind |  | HTH_6 | Ets |
|  | GATA | HTH_AraC | LytTR |  | HTH_8 | Fe_dep_repress |
|  | GerE | HTH_DeoR | MarR |  | HTH_AraC | Fez1 |
|  | GntR | KilA-N | Ogr_Delta |  | HTH_DeoR | FlhC |
|  | HLH | LacI | PadR |  | KilA-N | FlhD |
|  | Homeobox | LexA_DNA_bind | PerC |  | LacI | Fork_head |
|  | HSF_DNA-bind | LytTR | Rrf2 |  | LexA_DNA_bind | FUR |
|  | HTH_1 | MarR | SirB |  | LytTR | GATA |
|  | HTH_11 | MetJ | Trans_reg_C |  | MarR | GerE |
|  | HTH_3 | PadR | zf-C2H2 |  | MerR | GntR |
|  | HTH_5 | PerC |  |  | MetJ | HLH |
|  | HTH_6 | Rrf2 |  |  | Mga | Homeobox |
|  | HTH_8 | Rsd_AlgQ |  |  | Ogr_Delta | HSF_DNA-bind |
|  | HTH_AraC | SfsA |  |  | PadR | HTH_1 |
|  | HTH_DeoR | SirB |  |  | PerC | HTH_11 |
|  | HTH_psq | Trans_reg_C |  |  | PspC | HTH_3 |
|  | KilA-N |  |  |  | Rrf2 | HTH_5 |
|  | LacI |  |  |  | Rsd_AlgQ | HTH_6 |
|  | LexA_DNA_bind |  |  |  | SfsA | HTH_8 |
|  | LytTR |  |  |  | SirB | HTH_AraC |
|  | MarR |  |  |  | Trans_reg_C | HTH_DeoR |
|  | Not1 |  |  |  |  | HTH_psq |
|  | PadR |  |  |  |  | KilA-N |
|  | PerC |  |  |  |  | LacI |
|  | Pou |  |  |  |  | LexA_DNA_bind |
|  | PspC |  |  |  |  | LytTR |
|  | RFX_DNA_binding |  |  |  |  | MarR |
|  | RHD |  |  |  |  | MerR |
|  | Rrf2 |  |  |  |  | MetJ |
|  | Runt |  |  |  |  | Mga |
|  | SART-1 |  |  |  |  | Not1 |
|  | SirB |  |  |  |  | Ogr_Delta |
|  | TEA |  |  |  |  | PadR |
|  | TF_AP-2 |  |  |  |  | PerC |
|  | Trans_reg_C |  |  |  |  | Pou |
|  | zf-BED |  |  |  |  | PspC |
|  | zf-C2H2 |  |  |  |  | RFX_DNA_binding |
|  | zf-C2HC |  |  |  |  | RHD |
|  | zf-C4 |  |  |  |  | Rrf2 |
|  | zf-C5HC2 |  |  |  |  | Rsd_AlgQ |
|  | zf-MIZ |  |  |  |  | Runt |
|  | zf-TAZ |  |  |  |  | SART-1 |
|  |  |  |  |  |  | SfsA |
|  |  |  |  |  |  | SirB |
|  |  |  |  |  |  | TEA |
|  |  |  |  |  |  | TF_AP-2 |
|  |  |  |  |  |  | Trans_reg_C |
|  |  |  |  |  |  | zf-BED |
|  |  |  |  |  |  | zf-C2H2 |
|  |  |  |  |  |  | zf-C2HC |
|  |  |  |  |  |  | zf-C4 |
|  |  |  |  |  |  | zf-C5HC2 |
|  |  |  |  |  |  | zf-MIZ |
|  |  |  |  |  |  | zf-TAZ |
|  |  |  |  |  |  | Zim |

**References:**

[1] Frohlich DR, Torres-Jerez II, Bedford ID, Markham PG, Brown JK. A phylogeographical analysis of the *Bemisia tabaci* species complex based on mitochondrial DNA markers. Molecular ecology. 1999;8(10):1683-91.

[2] Tang YF, He Z, Du ZG, She XM, Lan GB. The Complex of Cotton leaf curl Multan virus and Its Associated Betasatellite Molecule Causing Cotton Leaf Curl Disease in Guangdong Province. Scientia Agricultura Sinica 2015;48(16):3166-75.
